# Behavior Analysis of Novel Wearable Indoor Mapping System Based on 3D-SLAM

**DOI:** 10.3390/s18030766

**Published:** 2018-03-02

**Authors:** Susana Lagüela, Iago Dorado, Manuel Gesto, Pedro Arias, Diego González-Aguilera, Henrique Lorenzo

**Affiliations:** 1 Department of Cartographic and Terrain Engineering, University of Salamanca, 05003 Avila, Spain; mgesto@usal.es (M.G.); daguilera@usal.es (D.G.-A.); 2 Applied Geotechnologies Research Group, University of Vigo, 36310 Vigo, Spain; idorado@uvigo.es (I.D.); parias@uvigo.es (P.A.); hlorenzo@uvigo.es (H.L.)

**Keywords:** wearable mapping prototype, SLAM, indoor mapping, LiDAR, accuracy analysis

## Abstract

This paper presents a Wearable Prototype for indoor mapping developed by the University of Vigo. The system is based on a Velodyne LiDAR, acquiring points with 16 rays for a simplistic or low-density 3D representation of reality. With this, a Simultaneous Localization and Mapping (3D-SLAM) method is developed for the mapping and generation of 3D point clouds of scenarios deprived from GNSS signal. The quality of the system presented is validated through the comparison with a commercial indoor mapping system, Zeb-Revo, from the company GeoSLAM and with a terrestrial LiDAR, Faro Focus^3D^ X330. The first is considered as a relative reference with other mobile systems and is chosen due to its use of the same principle for mapping: SLAM techniques based on Robot Operating System (ROS), while the second is taken as ground-truth for the determination of the final accuracy of the system regarding reality. Results show that the accuracy of the system is mainly determined by the accuracy of the sensor, with little increment in the error introduced by the mapping algorithm.

## 1. Introduction

The generation of 3D models and maps in building interiors is a task of increasing interest in different fields, from accessibility to energy efficiency and assistance in navigation through big buildings such as hospitals and commercial centers. This task is known as Simultaneous Localization and Mapping (SLAM) [1]. The first approaches in indoor navigation were in two-dimensions, and were dedicated to mobile robots, towards their autonomous movement [2]. Although the first robots were dedicated to movement indoors, medical applications appeared soon, incorporating the requirements of reduction of sizes for sensors and electronics [3] and adaptability to the human body. In this way, wearable devices were developed, with the main aim of contributing to mobility of humans with disabilities but also providing information on their positioning [4]. Some of these cases require an initial plan of the scenario [5], while others perform the mapping operation during displacement [6]. Among these systems, many are based on RGB-D cameras [7] or LiDAR sensors [8], given that these sensors directly provide the third dimension of space required for a proper navigation and mapping. The incorporation of an Inertial Measurement Unit (IMU) to provide more information about the movement depends on the platform and the algorithm used [9,10], while Global Navigation Satellite System (GNSS) is not operative for indoor scenarios. Regarding data processing, it is mostly based on SLAM algorithms, and Robotic Operative System (ROS) [11] for point cloud registration and map extraction, such as Cartographer Library from Google [12] and its modifications towards the 3D reconstruction of the scenes [13].

The products of current indoor mapping systems are 3D point clouds, for which accuracy tests are not yet extended and generalized as in the case of 2D maps [13]. Existing accuracy tests for 3D point clouds can be classified mainly into three approaches [14], according to the typology of the features selected for the study. These approaches are, in order of increasing accuracy: the control point approach, the subset approach, and the full point cloud or point cloud to point (p2p) cloud approach. The first consists of the evaluation of distances between corresponding point pairs in the point clouds under study; this way, a specific number of measurements stands for the whole point cloud. The subset approach takes a group of points from each point cloud under study (new and reference) and evaluates the differences between them, such as distance and deviations between planes. Last, the p2p methodology evaluates the two point clouds in their entirety; this approach provides more complete information about the quality of the point cloud in its three dimensions than the other approaches. These approaches focus on the point clouds generated from the static or mobile systems for indoor mapping, but no specific measurements regarding the deformation introduced by deviations in the trajectory are involved, which is a key factor regarding quality of the products for indoor mapping systems and the evaluation of the SLAM algorithm applied.

The evaluation of the capabilities of the indoor mapping system to track the poses in addition to the point cloud is a complex task, given the irregularity of the human displacement compared to a programmed robot, which can increase due to the fatigue induced from carrying a portable mapping system for a period of time. In the field of robotics, the evaluation of the pose computation is performed through the establishment of a compulsory trajectory for the robots and their tracking with different sensors [15,16]. Such accuracy is not necessary for indoor mapping systems, since partial deviations in the trajectory can be accepted if they do not affect the final point cloud.

In this paper, we present a wearable backpack-based indoor mapping prototype developed at the University of Vigo. It consists of 3D LiDAR and SLAM algorithms using ROS. An integral evaluation methodology is developed for the determination of the accuracy of the wearable prototype, combining the control point and the p2p approaches. In addition, the subset approach is applied for the indirect evaluation of the trajectory through the analysis of the shape of the point cloud in areas where the trajectory deviations could have an important effect, such as in the entrances in new spaces involving turns. With this aim, the wearable prototype is compared to both mobile and static commercial systems, Zeb-Revo [17] and Faro Focus^3D^ X330 [18]. The first is a mobile system consisting of a 2D LiDAR and an Inertial Measurement Unit (IMU) that spin around the horizontal axis in order to acquire information of the three-dimensions during displacement of the carrier operator. The generation of the point cloud is also performed using a SLAM algorithm with acquisition also controlled using ROS, and the most important difference regarding the wearable prototype is that the latter does not count with the presence of the IMU to help in the computation of the trajectory and the 3D point cloud with the 3D LiDAR data. The good performance of Zeb-Revo has already been established [19], in such a way that the point clouds generated are considered as reference in this paper regarding the maximum quality to achieve nowadays with mobile devices. The second device is a static terrestrial LiDAR, with different technical characteristics but better performance regarding point precision than both mobile systems (1 mm vs. 30 mm). In this way, they are considered as reference or ground-truth to examine the capacity of the wearable prototype to model the constructed reality.

Thus, the paper is organized as follows: Section 2 presents the wearable prototype, its configuration and algorithm development for point cloud generation. Section 3 includes the results obtained after the measurement of four case studies, chosen for their different configurations and difficulties, and the evaluation of the causes for the differences between case studies and systems. This is performed through an integral methodology including the control point and p2p approaches as well as an indirect evaluation of the trajectory through the measurement of its effect on the point cloud. Finally, Section 4 concludes the paper.

## 2. Materials and Methods

This section includes the description of the indoor mapping wearable prototype designed in the University of Vigo, and the systems used for the analysis of its performance.

### 2.1. Wearable Indoor Mapping Prototype from the University of Vigo

**Figure 1:**
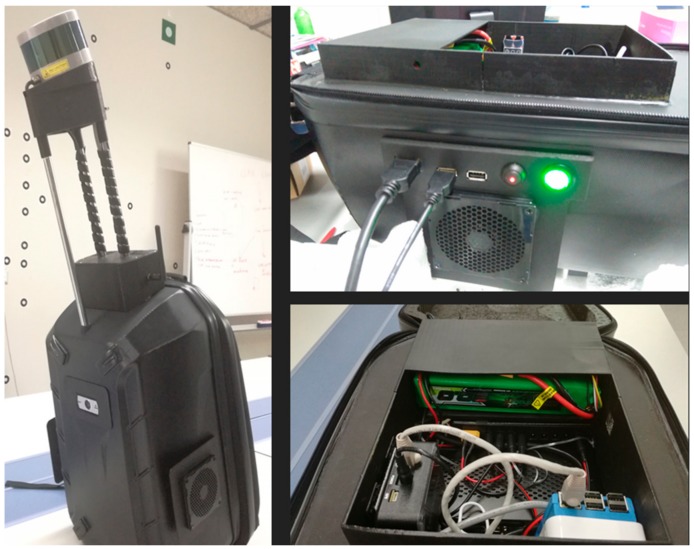
(**Left**) General view of the wearable prototype. (**Right**) Detail of the interior of the wearable prototype.

The wearable prototype presented in this paper consists of a platform, accessories for sensor integration, interior structures for the electronics, and the sensors themselves (Figure 1).

The platform selected is a backpack made of rigid plastic, and filled with hard foam, so that the electronics are firmly and safely stored. The inner structure presents a space in the centre, where the electronic components needed are fixed. These components include a compact computer board for the connection and control of the sensors, and the respective connection ports (ethernet) for communication and power supply. The battery connection is included in the interior of the wearable prototype, in such way that access is fast and easy for replacement purposes.

In addition, a set of serial ports (USB) and the on/off buttons for the control computer board and the 3D LiDAR sensor are embedded on the left side of the platform, providing full control of the system during measurement without need for opening (Figure 1-right). The serial ports are destined to the connection of the front camera and to data extraction via USB memory from the control computer board to the processing computer. The sides are also equipped with fans that keep the electronics under normal temperature during operation.

An external structure made of stainless steel and plastic is installed on the wearable prototype for the 3D LiDAR sensor (Figure 1). The objective of the structure is to place the sensor over the head of the operator and avoid occlusions in the data that its own body may cause. In addition, an increase in the height of the 3D LiDAR sensor ensures the acquisition of points both from the ground and the ceiling, provided that it only measures in a range of 30° vertically (Table 1). This sensor is a Velodyne VLP-16, which has been chosen due to its capability of acquiring 3D point clouds and its low weight and dimensions. In each scan, the sensor acquires the 3D coordinates of the points reached by the 16 rays of the 3D LiDAR, providing a sparse 3D point cloud for each position of the platform. In addition, two webcams are installed in the platform, one frontal and one in the back, with the aim of providing visual information of the environment for inspection purposes.

All the information measured is stored in the control computer board and can be extracted after each measurement for processing. The measurement is also controlled by the control computer board, as follows: the 3D LiDAR performs measurements and sends them to the control computer board, where each measurement is stored with the computer-time in the name of the file. This way, the incoming data is time-stamped and stored in the same file, in a binary file for acquisition structured and codified by ROS, whose corresponding format is “bag” [20,21]. This format is chosen due to the fact that it is the standard for data exchange between software developers and ROS. In addition, it allows for the reproduction of the acquisition and its postprocessing by manipulating the reproduction speed for the generation of more accurate results than when processed in real time.

**Figure 2:**
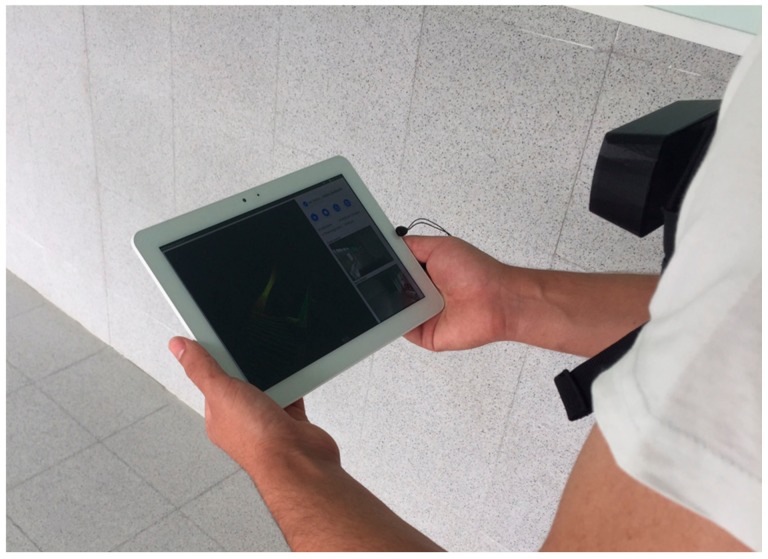
Image of the operator during inspection, with the wearable prototype controlled through the tablet.

Launch and control of each acquisition project is performed by the operator of the wearable prototype through a tablet (Figure 2), in such way that opening the prototype in the inspection site is not needed. The tablet is connected to the control computer board through a local Wi-Fi, that can also be used for other users within the Wi-Fi range to see the progress of the inspection.

One of the requisites during the design of the system is that acquisition should be performed in a straight-forward manner, in such a way that each room and corridor needs to be traversed only once to be correctly modelled in the point cloud. This stands in contrast with the procedure of most mobile indoor mapping systems, that require a ring-shape trajectory to ensure correct modelling of the environment; that is, the acquisition should start and finish in the same position, and each room should be traversed with a circular trajectory. By avoiding the necessity to perform ring-shaped acquisitions, the time required for inspection reduced by half, which is especially useful in large or long buildings such as academic buildings and commercial centers.

Tests are being performed to determine the best way to enter new rooms (frontally, laterally or backwards), with no determinant results about an optimal methodology for the wearable prototype. This stands in contrast with the methodology of the Zeb-Revo, which requires the entrance in new rooms laterally or backwards. This lack of variability in the results of the wearable prototype is due to the position of the 3D LiDAR and its acquisition of points in the 360° horizontally, that allows the acquisition of points from the new and the old rooms from the door without specific movements or changes in the operator’s position.

**Figure 3:**
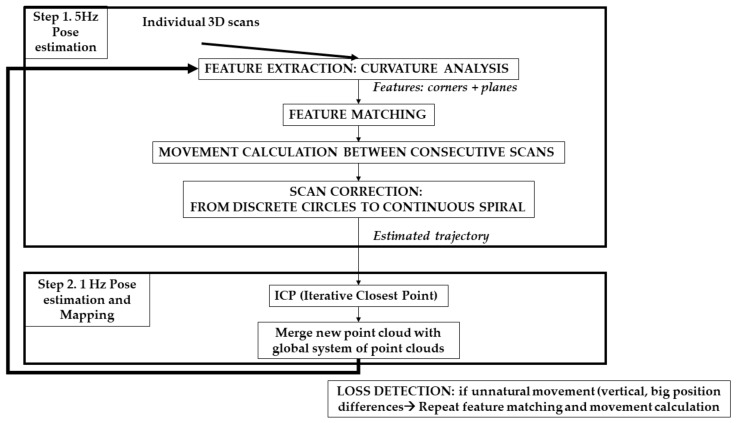
Pseudocode of the SLAM (Simultaneous Localization and Mapping) algorithm.

Data (point clouds acquired within the trajectory) are processed with an internally developed software, denominated as SLAM Post-process, and based on ROS, which is installed on a complementary computer, external from the backpack structure. The SLAM algorithm is performed in two steps, as detailed in Figure 3. The first step consists of the estimation of the trajectory and the correction of the movement. To do this, the individual 3D scans acquired are subjected to an iterative process at high speed, with a frequency of 5 Hz (that is, the scans introduced in the process are those acquired with a 5 s interval: among all the scans acquired, only 1 scan every 5 s is subjected to registration and used for the coarse estimation of the trajectory). A curvature analysis is performed to the scans for the extraction of two types of features: angular features such as corners and columns, and planar features. These features are extracted through a curvature analysis, based on the nearest closest points. Each neighborhood of points is used for the computation of the normal vector of each point through Principal Components Analysis (PCA) in which those points with a normal vector different from the four closest points in the neighborhood are identified as angular features, and those points sharing the same direction of the normal vector as at least 20 points in the neighborhood are classified as planar features. These thresholds are established as a compromise between the availability of information to avoid the identification of outliers as features and the minimization of points used to manage the computation at 5 Hz.

The features are submitted to an iterative matching process, which is performed per line (or ray) of the scan, as it is an extrapolation of the procedure performed for 2D LiDAR sensors that only measure one line per acquisition through repetition of the procedure 16 times, one per ray or channel of the Velodyne sensor integrated in the mobile platform. This coarse registration of point clouds allows for the extraction of the estimated trajectory as well as an estimation of the rotation matrix and translation vector between the scans. With this estimation, the theoretical circular form of the individual scans is corrected and transformed into a continuous spiral, which is a more realistic representation of the scanning process during movement and allows for a better estimation of the posterior scan registration and trajectory refinement.

In the second step of the SLAM processing, all the points acquired in the individual scans are used in a registration process based on Iterative Closest Point (ICP) algorithm [22,23]. This registration is performed at 1 Hz speed; to ensure this frequency, the registration is performed for the scans acquired within 5 s intervals. This time interval does not affect the precision of the final result since the computation of the registration is helped by: (1) the estimated trajectory, that provides a preliminary position of the individual scans related to the trajectory; (2) the estimation of the transformation between scans (rotation matrix and translation vector) and (3) the corrected spiral form of the scanning during movement.

The registration of individual point clouds during SLAM is complemented with an algorithm for the automatic detection of losses in the trajectory. This algorithm detects the appearance of sudden and unrealistic changes in the computation of the trajectory, such as vertical movements, jumps, and big distances between contiguous positions, based on the assumption that these movements are not a realistic performance of a human operator. Once a loss is detected the algorithm stops the computation of the trajectory and registers a buffer of first lost positions with a buffer of positions in the part of the trajectory assumed as correct. For this process, Statistical Outlier Removal (SOR) Passthrough algorithms are applied to eliminate points measured in each pose with a great distance from the center of the point cloud and consequently present low accuracy, in addition to an MLS Moving Least Squares (MLS) filter to improve the shape of the point cloud while eliminating points associated to noise. Once the pose is recalculated, the SLAM algorithm is restarted.

**Figure 4:**
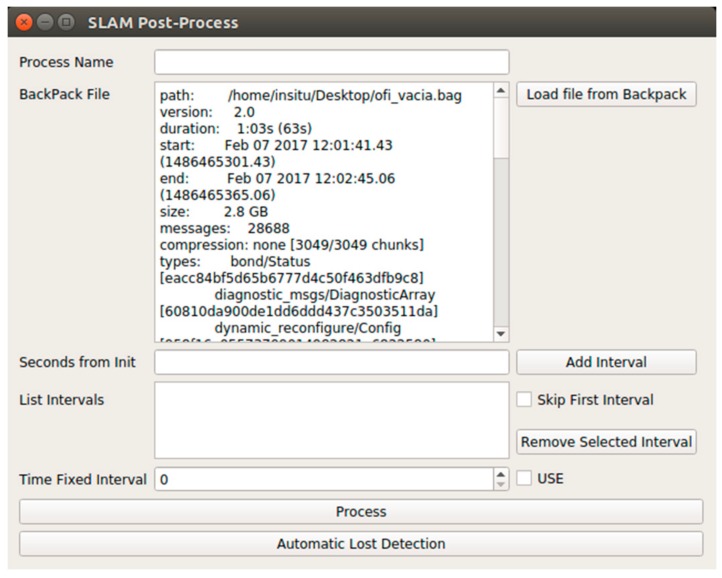
SLAM configuration window, for the definition of processing intervals and the determination of the steps of the process to automate.

The application of the SLAM algorithm can be configured prior to start (Figure 4), including the definition of intervals for partial processing and the decision of application of Loss Detection, which is optional.

**Figure 5:**
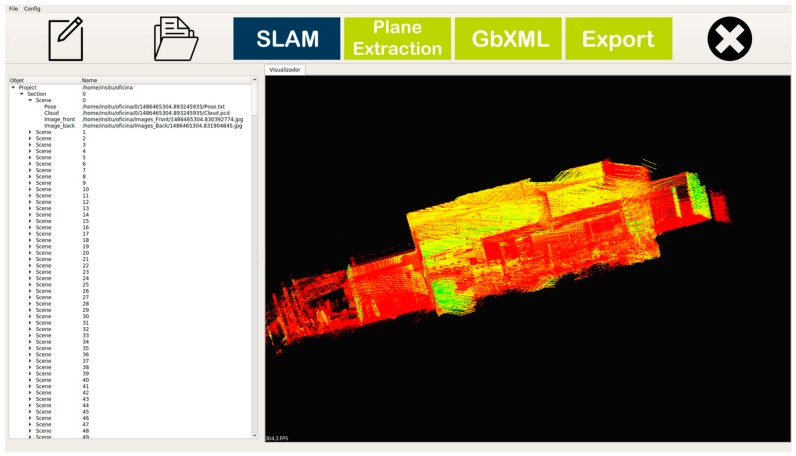
Main interface after SLAM. The composition tree at the left includes the individual point clouds associated to each pose of the computed trajectory.

Once the SLAM is finished and the 3D point cloud of the building interior is generated, the point cloud is stored in a tree-like structure, where the poses of the trajectory store the coordinates of all the points measured from them. Thus, the tree of the composition of the point cloud is loaded to the main interface (Figure 5), allowing the selection of single scenes for visualization purposes. These scenes are composed by the individual point clouds and an image pair from the webcams. In the case that more than one image pair is available for a given position in the trajectory computed, the focus level of the images is analyzed, and the image pair with less blur is associated to the position dataset.

### 2.2. Commercial Systems

**Figure 6:**
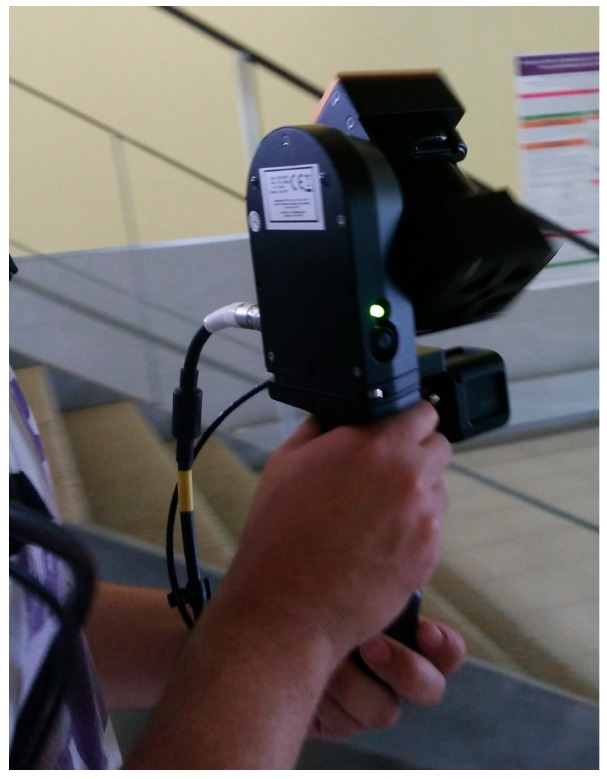
View of the Zeb-Revo during acquisition.

The commercial mobile mapping system Zeb-Revo, from the company GeoSLAM, is used for the measurement of the same scenarios as the wearable prototype. It is a second version of their system for indoor mapping, whose first version was named Zebedee [24]. While the previous version was formed by a 2D LiDAR moved with a spring, the Zeb-Revo consists of a hand-held platform equipped with a 2D LiDAR, Hokuyo UTM-30LX-F, that rotates during measurement (Figure 6). This laser scanner is a special version of Hokuyo which acquires at higher frequency rates a lower number of points than the commercial version; in this way, the number of points is sacrificed towards the increase in the number of scenes acquired, thus minimizing the risk of losing the pose calculation due to the fast inspection of the new positions. In addition, an IMU is placed on top of the 2D LiDAR in order to control its position during rotation and displacement of the operator. Thanks to the rotation, the system acquires information of the three-dimensions of the area under study, enabling the application of 3D-SLAM algorithms using a 2D LiDAR. Sensors and data acquisition are controlled by a PC which is stored in the backpack that should be worn by the operator during inspection. In addition to communication, the backpack includes batteries to power the handheld platform, both tasks performed through the corresponding connection cables. Technical characteristics are shown in Table 2.

Regarding data acquisition, the main characteristic of the Zeb-Revo system is that inspections should start and finish in the same point and position, and trajectories should present ring-shape. In addition, when entering and exiting new rooms, the system should be laterally turned, so that, at the door, information is acquired from both the old and the new room.

The case studies are also measured with the terrestrial laser scanner FARO Focus^3D^ X330, which has been chosen as ground truth due to its high precision and accuracy in the measurements, as stated in Table 3.

The comparison between methodologies requires that all point clouds represent the same areas. While Mobile Mapping Systems have the capacity to measure large areas with no direct visual contact between them, the acquisition of point clouds from different positions is required for a methodology based on terrestrial LiDAR. The scans acquired with FARO Focus^3D^ X330 for each case study are registered to the same coordinate system resulting in a unique point cloud representing the complete area covered by the Mobile System. Registration is performed with Iterative Closest Point algorithm (ICP) [22,23], starting from a coarse registration with 3 corresponding points and followed by a fine registration using all points in the point clouds and the coarse registration results.

### 2.3. Case Studies

Different case studies have been analyzed with the three systems (wearable mapping prototype, Zeb-Revo and Faro Focus), selected for their representability of common indoor scenarios:

Case study 1: corridor with doors and entrances at both sides, presenting symmetrical similarity and a repetitive pattern. This scenario involves a complex case study for the mobile systems based on SLAM due to the high probability of identifying two consecutive features as the same one given the lack of differentiation in their environments, and consequently obtaining a repetition of results for pose estimation, meaning null displacement between estimated poses.

Case study 2: big square hall, in which two walls are composed by glass windows. This scenario implies a difficulty to all scanning systems in general due to the reflectivity of glass, that provokes the appearance of a double registration for the points in the windows. The dimensions of the hall make it difficult for the mobile systems to extract features homogeneously distributed within the scene for a correct SLAM execution. In addition, the hall constitutes the entrance to the building, so the presence of people moving is unavoidable, with the consequent appearance of artifacts in the point cloud. Furniture such as tables, benches, standing posters and a vending machine have not been moved in order to test the performance of the system with elements that provoke occlusion.

Case study 3: corridor-room system, constructed with materials such as bricks and concrete. This case study presents no special requirements regarding material reflectivity but is chosen to study the capability of the system for scene reconstruction including turns and entrances in new spaces.

Case study 4: corridor-room system, where one side wall of the corridor is composed of glass material, and the corridor presents a repetitive layout. Thus, in addition to the complexity for reconstruction introduced by the presence of reflections in the wall, the possibilities for the systems to lose track of the displacement are evaluated.

The number points of each acquisition are shown in Table 4. The measurement with FARO Focus was limited to two scan positions in order to equal the time needed by the mobile indoor mapping systems for inspection.

In order to perform an analysis of the performance of the systems under real circumstances, the presence of people in the study areas was allowed and cluttering and occlusions were present since no furniture was moved from its original position.

## 3. Results and Discussion

The point clouds generated with the different system for the different case studies are analyzed in several ways: first, a visual evaluation is performed for the analysis of the data directly obtained with the sensors, and the identification of sources of noise and artifacts in the point clouds. Second, a 2D geometrical evaluation is performed through 2D sections and with features such as distances between points in order to evaluate the shape of the point clouds generated. Last, a point to point analysis is performed after the registration of the point clouds of each case study to the same coordinate system, with the aim at computing deviations in the generation of point clouds from the mobile devices, including possible angular deviations appearing with turns in the trajectory.

### 3.1. Point Clouds

The point clouds acquired with the different systems in the four case studies are shown in Table 5. In these images, the higher appearance of points corresponding to noise can be noticed for the indoor mapping systems, with slight difference among them. For all case studies, the main cause of noise is the presence of windows and glass surfaces, that corrupts the measurement and provokes the reflection of the laser rays and the consequent appearance of double points in the point cloud [25]. The effect of glass surfaces can be especially seen in Case study 4, where the left wall of the corridor is doubled (considering the room is at the right side of the corridor). This is also the cause for the angular point groupings projecting from the point clouds. Case studies 3 and 4 are only measured with the mobile indoor mapping systems because the purpose of their choice is to evaluate the deviation introduced by each SLAM methodology in the measurement of long and straight measurements (longer than 100 m).

Regarding the two indoor mobile mapping systems, the point clouds from the wearable mapping prototype present more points belonging to noise than the Zeb-Revo. There could be three reasons for this: one is the higher number of points acquired per scan of the wearable mapping prototype through its 16-ray device and the capability of measuring their echoes, while the Zeb-Revo acquires points with only one ray that rotates, and its laser scanner is specially designed to acquire less points per ray to have a higher acquisition rate. This fact can also be noticed in the smaller number of points of the Zeb-Revo in comparison with the wearable mapping prototype in Table 5. The second reason is software-related: although both systems generate point clouds with SLAM algorithms based on ROS, they present different parameters regarding the percentage and the distance of points to include in the computations and thus the number of points included in the final point cloud. An increase in the number of points can lead to the appearance of more points associated to noise, but it also allows a more robust computation of the point cloud registration especially in scenarios with big dimensions where key points for registration are far from the beginning of the mapping: this is the case in Case studies 1 and 4, where the structure of the corridor is repetitive and would provoke the loss of the position in the trajectory if there are no points measured in the surfaces of both the beginning and the closure of the corridor. Last, the position of the laser scanner in the wearable mapping prototype, although being optimal to maximize the measurement of useful points and avoid a reduction in the acquisition angle due to the presence of the operator, implies the angular incidence of some rays in the environment, favoring their deflection and thus the appearance of points corresponding to noise.

### 3.2. 2D Analysis

Given that one of the most extended uses of indoor mapping systems is the generation of 2D maps for navigation purposes, the accuracy of the 2D measurements in the point clouds generated by the mobile systems has been evaluated as follows. In order to ensure the two-dimensionality of the measurements, a horizontal section has been extracted from each point cloud by selecting those points with *z*-value of 1.5 ± 0.1 m; with this value, the presence of furniture occluding the walls is limited to wardrobes and shelves, avoiding chairs and tables. In addition, in order to check the behavior of the wearable prototype in the two directions determining displacement: longitudinal and perpendicular to the trajectory, measurements have been grouped according to them.

One of the first interpretations introduced by the results is the higher error in the direction of displacement of the system, which is common for both mobile systems (Table 6). The reason is that this dimension is a result of the computation of the trajectory, while the perpendicular dimension is the result of the direct measurement of the laser scanner device. The latter is reinforced by the agreement between the error in the direction perpendicular to the trajectory and the nominal precision of the sensors.

However, Case study 2 shows an opposite distribution of the results (Table 7). The explanation for this change is the dimensions and shape of the case study, more quadrangular than the other case studies. This allows for the longitudinal dimensions to be measured also perpendicularly to the displacement, following a path in a ring-form in the central area of the case study. Thus, these measurements (hall length and width) are performed both along displacement and directly by the laser scanner sensor, in such a way that their accuracy is double regarding points measured only in one direction. The same happens for the measurement of the small room.

Case studies 3 and 4 are subjected to the analysis of the difference between the point cloud of the wearable mapping prototype and the Zeb-Revo, for the sake of comparison between mobile systems (Table 8 and Table 9, respectively).

For all case studies, the higher error in the wearable mapping prototype is found in the measurement of the smallest dimensions. The reason for this is the difficult clear identification of the borders in the point clouds from this system due to the presence of noise and the high number of points. An example of this effect is the impossibility for detecting Entrance 1 in Case study 1 (Table 7, Figure 5). Noise and point reduction could be obtained through the filtering of the point cloud, but it also implies the loss of spatial resolution, so the configuration of the filtering process should be performed as a compromise.

In addition, the highest error among all case studies is obtained for Case study 2 for both mobile systems, in such a way that the low performance of the systems can be associated to the characteristics of the case study, such as the presence of a main wall made of glass and the existence of numerous obstacles and people moving within the study area, leading to the appearance of artifacts and moving points in the measurements that introduced complexity in the point cloud registration for SLAM.

### 3.3. p2p Analysis

The point clouds of each case study are registered into the same coordinate system through the automatic detection and matching of corresponding intensity features (artificial targets placed homogeneously in the scenarios of the case studies), establishing the Zeb-Revo point cloud as reference for mobile systems. The errors of these registrations (after ICP) are shown in Table 10.

Point-cloud to point-cloud distances are computed based on a previous kd-tree division of the two point-clouds under analysis in order to determine the nearest neighbor point in one point cloud of each point in the reference point cloud (k = 1) for distance calculation [26]. Case study 2 shows the distance between the ground-truth and both mobile systems under study. Point clouds from the wearable mapping prototype and the Zeb-Revo have shown a mean distance with the point cloud of the FARO Focus, considered as ground-truth, of 0.11 ± 0.23 m and 0.11 ± 0.34 m, and mode distances (most populated distances) of 0.19 m and 0.03 m, respectively. The high value of the deviations (0.23 m and 0.34 m) is produced by the presence of more points in the scenes from the mobile devices, most of them corresponding to noise and increasing the error computed (Table 5 and Table 6). With these results, the Zeb-Revo shows higher closeness to the ground-truth and is thus chosen as reference for the analysis of the point cloud of the wearable prototype for the other case studies (Table 11 and Table 12).

According to these results, the general accuracy is in agreement with the technical characteristics of the laser scanner sensor, given that the mean distance is close to 0.06 m for Case studies 3 and 4, which is within the point precision of ±0.03 m for the LiDAR sensors in both systems (Velodyne in the Wearable Mapping Prototype and Hokuyo in Zeb-Revo, Table 1 and Table 2). In addition, the presence of longer distances associated with larger error values for Case studies 1 and 4 (55 m and 110 m, respectively, compared to the longest distances of 24 m and 46 m in case studies 2 and 3) leads to the conclusion that the wearable mapping prototype does accumulate deviation with the trajectory, since these case studies present the largest distances and corridors. This deviation could be reduced (to be tested) through the implementation of ring-like trajectories if the time requirements of the inspection allow for it, as in the case of the Zeb-Revo, in order to have a double validation of distance measurements and make possible the detection of loop closure and graph optimization. Another option would be the implementation of a loop closure strategy through the identification of image descriptors in the images of the webcams in the wearable mapping prototype. Image features would make up a dataset independent from the errors performed by the SLAM algorithm and consequently with higher possibilities for error detection and reduction. This way, the error in the adjustment could be corrected without the need of duplicating the number of point clouds.

In this analysis, Case study 2 does not appear as critical, since both systems are mobile and are affected by the presence of the furniture and people for the resolution of the SLAM.

## 4. Conclusions

The Wearable Mapping Prototype for indoor mapping developed in the University of Vigo has been presented and tested in this work, through the comparison of results with the results from a commercial indoor mapping system and a terrestrial laser scanner. The validation has been performed through an integral methodology including linear 2D features and point cloud to point cloud analysis. In this way, both the mapping and the capacities for 3D modelling are evaluated.

Results show that the performance of the Wearable Mapping Prototype is comparable with the performance of the commercial indoor mapping system, with the advantage that the Wearable Mapping Prototype presents room for further improvement through the future integration of data from an IMU and the detection of positions revisited within the trajectory, while maintaining the lack of constraints for the design of the trajectory of inspection.

The accuracy in the 2D measurements is within the technical characteristics of the laser scanner integrated in the platform for each mobile system (Wearable Mapping Prototype and Zeb-Revo), and the point cloud to point cloud accuracies are similar for both cases, with mean distances of 0.1 m with the ground truth.

The main dissimilarity between the point clouds of both mobile systems is the higher number of points in the Wearable Mapping Prototype; the hardware and software reasons have been identified and analyzed in the paper to reveal the main difference between the SLAM methodologies of the systems. While the presence of a high number of points can be inconvenient for visualization and imply higher needs for processing towards the modelling of the building, it also implies a higher detail for the modelling of furniture and other elements present in the scene.

Thus, the Wearable Mapping Prototype, in spite of the detection of improvements of interest, has shown its validity for the mapping of indoor scenes according to the current capacities of technology.

## Figures and Tables

**Table 1:** Technical characteristics of Velodyne VLP-16 provided by the manufacturer.

Velodyne VLP-16	
Weight	800 g
Laser rays	16 channels
Range	100 m
Acquisition rate	300,000 points per second
Point accuracy	3 cm
Field of view	360° (H) × 30° (V)

**Table 2:** Technical characteristics of the Zeb-Revo portable mobile mapping system provided by the manufacturer.

Technical Characteristic	Value
Measurement range	30 m (indoors)/15 m (outdoors)
Measurement speed	43,200 points/second
Accuracy in the 3D representation	±0.1%
Field of view	270° (H) × 360° (V-in rotation)
Weight of head scanner	1.0 kg

**Table 3:** Technical characteristics of terrestrial LiDAR FARO Focus^3D^ X330 provided by the manufacturer.

Technical Characteristic FARO Focus^3D^ X330	Value
Measurement range	0.6 to 330 m
Measurement speed	Up to 976,000 points/second
Accuracy (for measurements of 10–25 m)	±2 mm

**Table 4:** Data acquisition per system and case study scenario (number of points).

	Wearable Mapping Prototype	Zeb-Revo	FARO Focus^3D^ X330
Case study 1	16,602,865	7,920,055	27,967,488
Case study 2	15,733,188	6,657,601	9,361,846
Case study 3	10,708,101	7,238,539	-
Case study 4	29,581,087	13,765,082	-

**Table 5:** Top view of the point clouds generated per system and case study. Differences in color are due to different measurement values of the intensity of the return signal of UVIGO Wearable Prototype and FARO Focus X330. For the case of Zeb-Revo, the representation is done through a pseudocolor according to the distance measured between the system and the object.

	UVIGO Wearable Prototype	Zeb-Revo	FARO Focus^3D^ X330
Case study 1	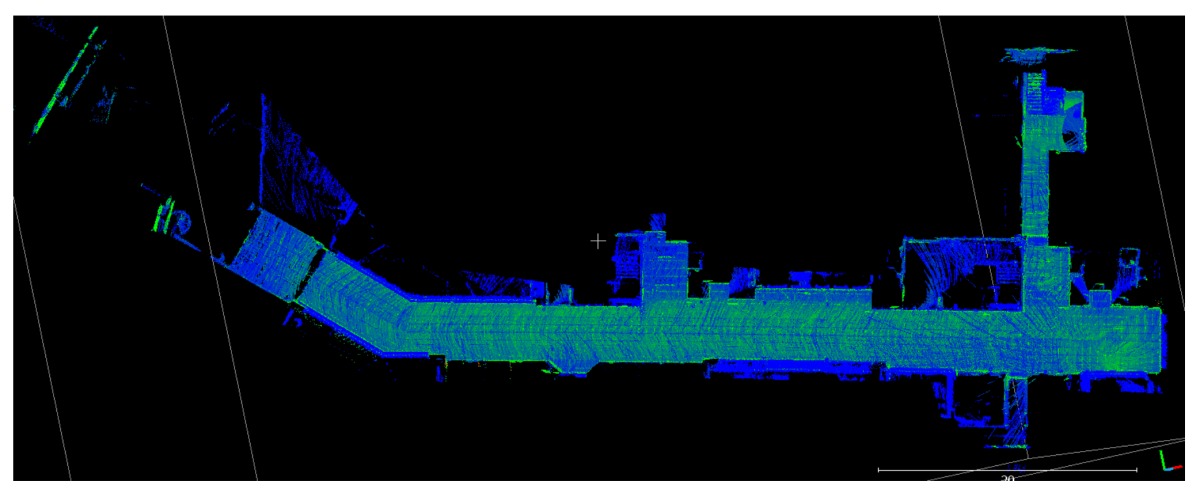	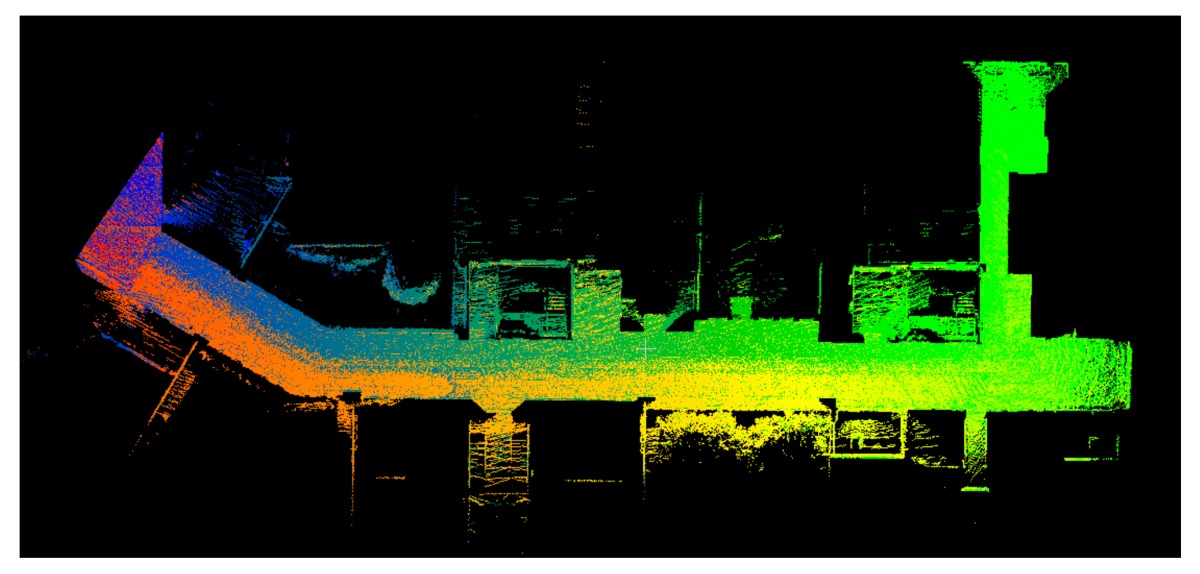	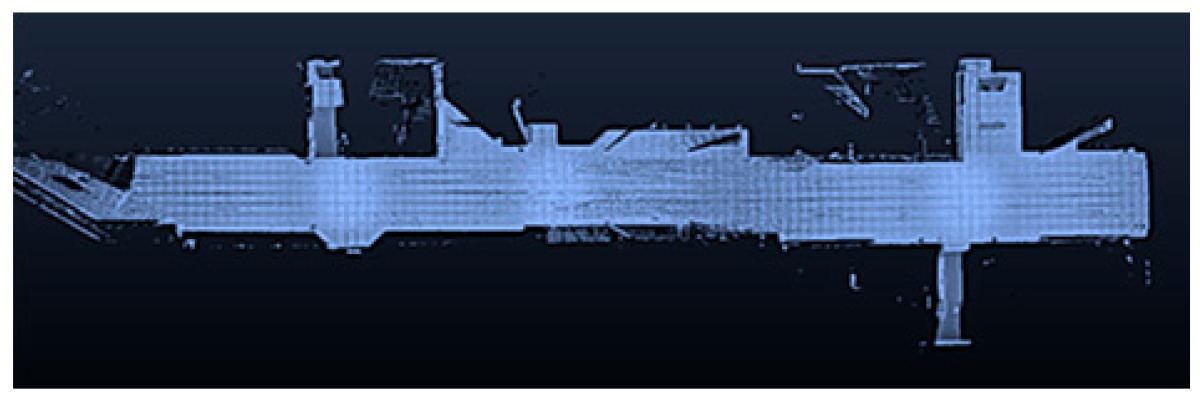
Case study 2	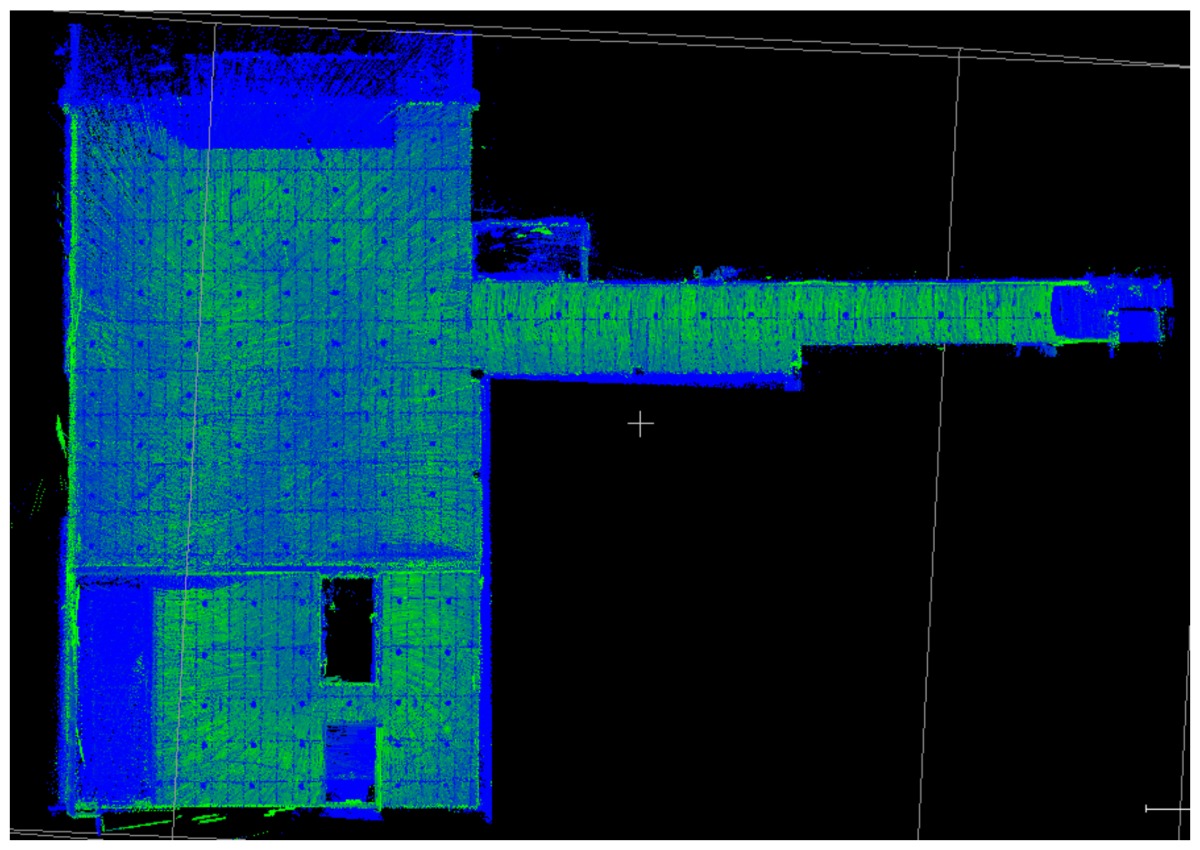	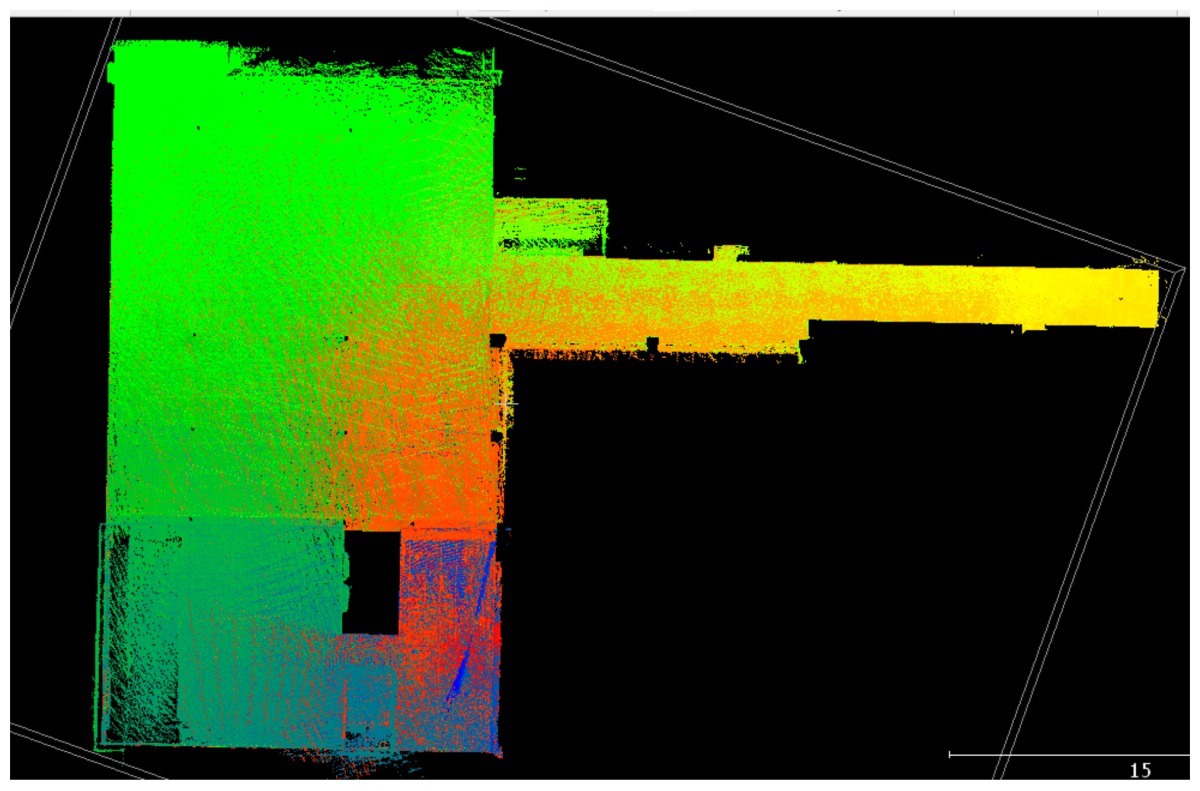	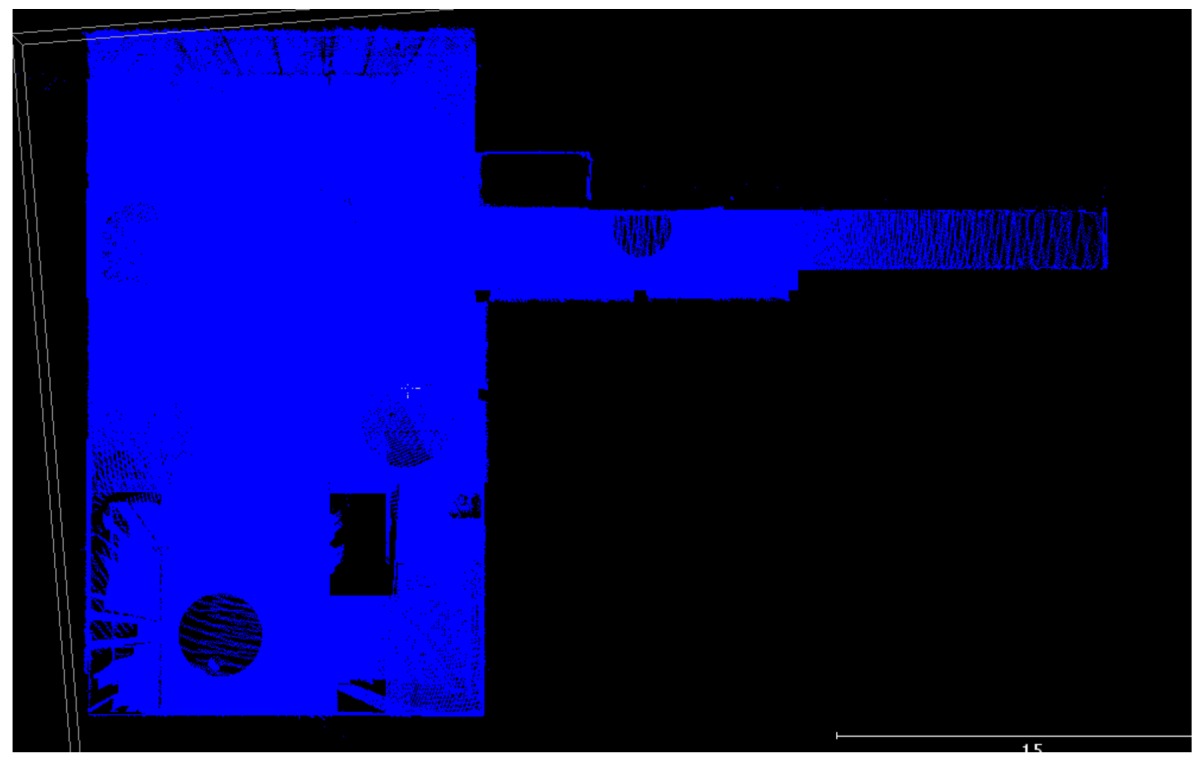
Case study 3	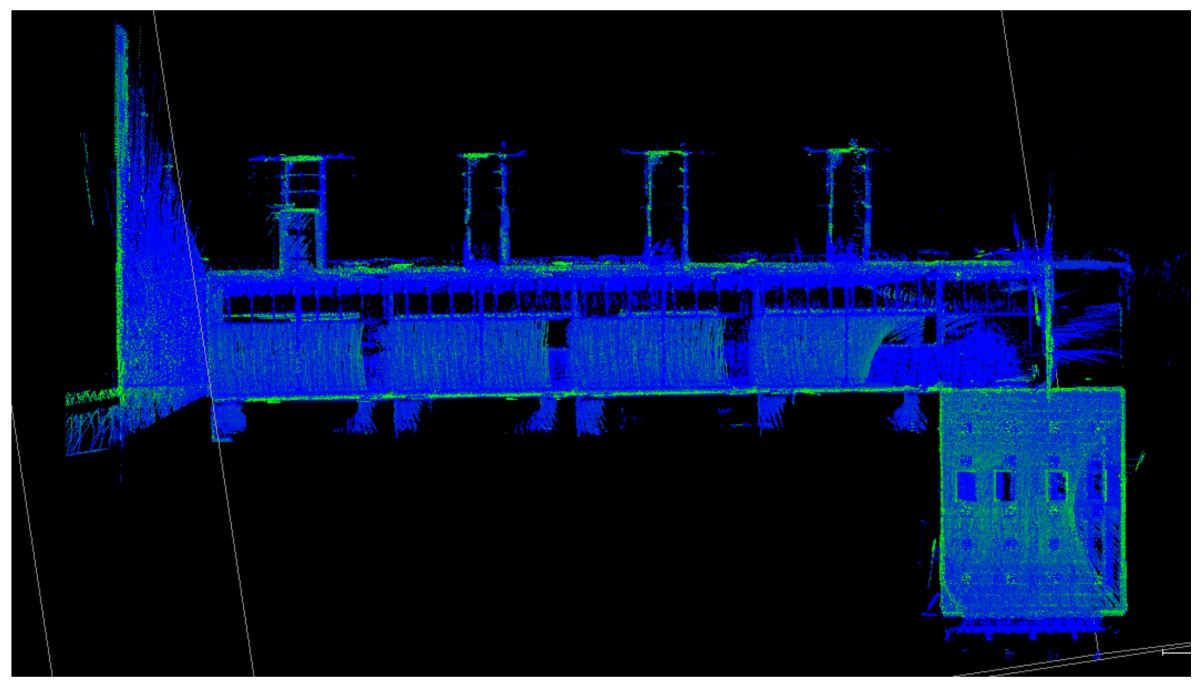	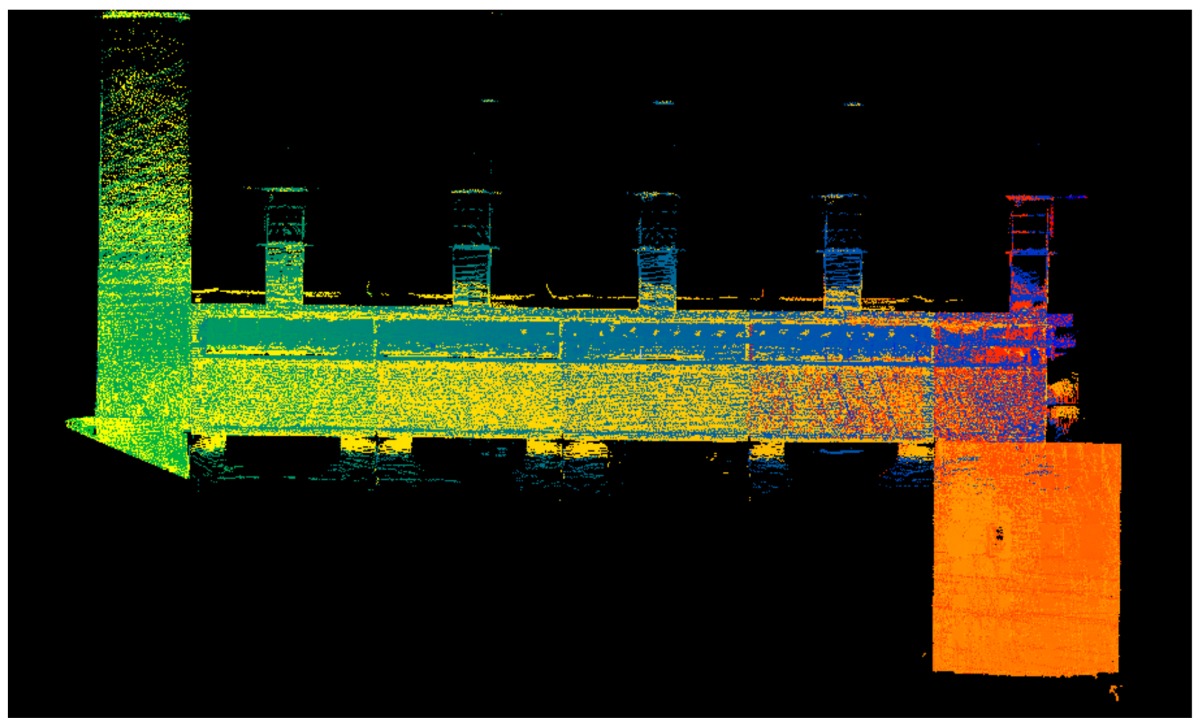	-
Case study 4	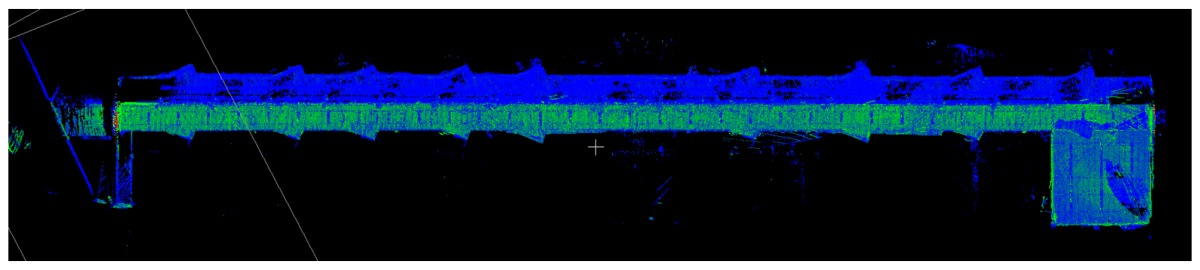	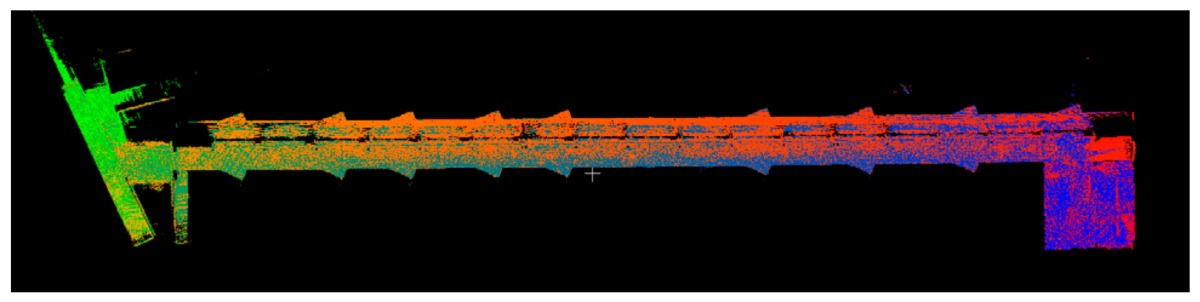	-

**Table 6:** Results of the 2D measurements for case study 1, in meters. “nd” is applied to non-distinguishable features. “WMP” represents the wearable mapping prototype designed and developed at the University of Vigo. “ZR” denotes Zeb-Revo commercial mapping mobile system.

Directon	Measur.	WMP	ZR	FARO	ERROR WMP = ABS (FARO-WMP)	%ERROR WMP = ERROR·100/FARO	ERROR ZebRevo = ABS (FARO-ZR)	%ERROR ZebRevo = ERROR*100/FARO
Longit.	Entrance 1	nd	1.63	1.72	-	-	0.09	5.23%
	Entrance 2	1.35	1.34	1.39	0.04	2.88%	0.05	3.59%
	Room 2	11.85	11.58	12.22	0.37	3.03%	0.64	5.24%
	Corridor Length	55.02	55.09	55.79	0.77	1.38%	0.70	1.25%
Perpend.	Corridor Width 1	4.29	4.39	4.35	0.06	1.37%	−0.04	0.92%
	Corridor Width 2	4.04	4.02	4.06	0.02	0.49%	0.04	0.98%
	Corridor Width 3	4.50	4.51	4.53	0.03	0.66%	0.02	0.44%

**Table 7:** Results of the 2D measurements for case study 2, in meters. Column 6 stands for Error of the wearable prototype (in the following, WMP), as the absolute difference between the distance measure in the point cloud from the FARO laser scanner and the WMP. Column 7 includes the relative error (in percentage) of the point cloud from the WMP, referred to the distance measured by the FARO as ground-truth. Columns 8 and 9 present the same absolute errors and relative errors, for the Zeb-Revo device (denoted as ZR).

Direct	Meas.	WMP	ZR	FARO	ERROR WMP = ABS (FARO-WP)	%ERROR WMP = ERROR·100/FARO	ERROR ZebRevo = ABS (FARO-ZR)	%ERROR ZebRevo = ERROR*100/FARO
Longit.	Corridor length	24.01	24.39	24.20	0.19	0.78%	−0.19	−0.78%
	Hall length	24.69	24.62	24.57	−0.12	−0.49%	−0.05	−0.20%
	Hall width	15.13	15.16	14.94	−0.19	−1.27%	−0.22	−1.47%
Perpend.	Corridor width 1	3.23	2.92	3.09	−0.14	−4.53%	0.17	5.50%
	Corridor width 2	2.19	2.09	2.13	−0.06	−2.69%	0.04	1.88%
	Room width	2.23	2.26	2.23	0.00	0%	−0.03	1.34%
	Distance wall-machine	3.56	3.37	3.23	−0.33	10.22%	−0.14	−4.33%

**Table 8:** Results of the 2D measurements for Case study 3, in meters.

Direct	Meas.	WMP	ZR	DIFFERENCE WMP = ABS(ZR-WMP)	%DIFF. WMP = ERROR*100/ZR
Longit.	Corridor length	45.74	45.77	0.03	0.06%
	Balcony length 1	5.06	5.12	0.06	1.17%
	Balcony length 2	9.85	9.90	0.05	0.51%
	Entrance	1.16	1.17	0.01	0.85%
Perpend.	Corridor width	6.51	6.48	−0.03	−0.46%
	Room length	9.85	9.77	−0.08	−0.82%
	Room width	12.06	11.94	−0.12	−1.00%
	Balcony width	1.86	1.91	0.05	2.61%

**Table 9:** Results of the 2D measurements for Case study 4, in meters.

Direct	Meas.	WMP	ZR	DIFFERENCE WMP = ABS(ZR-WMP)	%DIFF. WMP = ERROR*100/ZR
Longit.	Corridor length	110.19	110.45	0.26	0.23%
	Entrance	0.70	0.73	0.03	4.11%
	Diagonal Entrance	2.90	2.75	−0.15	−5.45%
Perpend.	Corridor width 1	2.60	2.57	−0.03	−1.16%
	Corridor width 2	3.70	3.45	−0.25	−7.25%
	Room length	9.78	9.78	0.00	0%
	Room width	10.12	10.18	0.06	0.59%

**Table 10:** Error in point cloud registration (RMSE). Units: m.

System Registered to Zeb-Revo Coordinate System	Case Study 1	Case Study 2	Case Study 3	Case Study 4
Wearable Mapping Prototype	0.085	0.088	0.086	0.116
FARO FOCUS	0.062	0.089	-	-

**Table 11:** Point-cloud to point-cloud distance between the Wearable Mapping Prototype, the Zeb-Revo and the FARO Focus, for Case study 2.

Comparison with FARO Focus Point Cloud		Wearable Mapping Prototype	Zeb-Revo
Mean distance (m)		0.107	0.107
Standard Deviation (m)		0.232	0.343
Most populated distance	Number of Points	12,848,493	7,698,907
	Distance (m)	<0.191	<0.030
Significant greatest distance	Number of Points	902	1540
	Distance (m)	3.048	0.989

**Table 12:** Point-cloud to point-cloud distance between the Wearable Mapping Prototype and the Zeb-Revo, for the four case studies.

Wearable Mapping Prototype vs. Zeb-Revo		Case Study 1	Case Study 2	Case Study 3	Case Study 4
Mean distance (m)		0.228	0.009	0.063	0.072
Standard Deviation (m)		1.652	0.501	0.177	0.0932
Most populated distance	Number of Points	15,916,124	15,189,412	8,991,535	26,496,495
	Distance (m)	<0.448	<0.377	<0.072	<0.078
Significant greatest distance	Number of Points	1158	857	834	1203
	Distance (m)	2.241	1.883	3.588	7.798
